# A Lipophilic Fucoxanthin-Rich *Phaeodactylum tricornutum* Extract Ameliorates Effects of Diet-Induced Obesity in C57BL/6J Mice

**DOI:** 10.3390/nu11040796

**Published:** 2019-04-06

**Authors:** Andrea Gille, Bojan Stojnic, Felix Derwenskus, Andreas Trautmann, Ulrike Schmid-Staiger, Clemens Posten, Karlis Briviba, Andreu Palou, M. Luisa Bonet, Joan Ribot

**Affiliations:** 1 Max Rubner-Institut, Federal Research Institute of Nutrition and Food, Department of Physiology and Biochemistry of Nutrition, 76131 Karlsruhe, Germany; andrea.gille@mri.bund.de (A.G.); karlis.briviba@mri.bund.de (K.B.); 2 Laboratory of Molecular Biology, Nutrition and Biotechnology, Universitat de les Illes Balears, 07122 Palma de Mallorca, Spain; bojan.stojnic@uib.es (B.S.); andreu.palou@uib.es (A.P.); joan.ribot@uib.es (J.R.); 3 Institute of Interfacial Process Engineering and Plasma Technology IGVP, University of Stuttgart, 70569 Stuttgart, Germany; felix.derwenskus@igb.fraunhofer.de; 4 Fraunhofer Institute for Interfacial Engineering and Biotechnology IGB, 70569 Stuttgart, Germany; ulrike.schmid-staiger@igb.fraunhofer.de; 5 Karlsruhe Institute of Technology (KIT), Institute of Process Engineering in Life Sciences III Bioprocess Engineering, 76131 Karlsruhe, Germany; andi.t@gmx.de (A.T.); clemens.posten@kit.edu (C.P.); 6 CIBER de Fisiopatología de la Obesidad y Nutrición (CIBEROBN), 07122 Palma de Mallorca, Spain; 7 Institut d’Investigació Sanitària Illes Balears (IdISBa), 07120 Palma de Mallorca, Spain

**Keywords:** *Phaeodactylum tricornutum*, microalgae, fucoxanthin, eicosapentanoic acid, obesity, browning, brown adipose tissue

## Abstract

*Phaeodactylum tricornutum* (*P. tricornutum*) comprise several lipophilic constituents with proposed anti-obesity and anti-diabetic properties. We investigated the effect of an ethanolic *P. tricornutum* extract (PTE) on energy metabolism in obesity-prone mice fed a high fat diet (HFD). Six- to eight-week-old male C57BL/6J mice were switched to HFD and, at the same time, received orally placebo or PTE (100 mg or 300 mg/kg body weight/day). Body weight, body composition, and food intake were monitored. After 26 days, blood and tissue samples were collected for biochemical, morphological, and gene expression analyses. PTE-supplemented mice accumulated fucoxanthin metabolites in adipose tissues and attained lower body weight gain, body fat content, weight of white adipose tissue (WAT) depots, and inguinal WAT adipocyte size than controls, independent of decreased food intake. PTE supplementation was associated with lower expression of *Mest* (a marker of fat tissue expandability) in WAT depots, lower gene expression related to lipid uptake and turnover in visceral WAT, increased expression of genes key to fatty acid oxidation and thermogenesis (*Cpt1*, *Ucp1*) in subcutaneous WAT, and signs of thermogenic activation including enhanced UCP1 protein in interscapular brown adipose tissue. In conclusion, these data show the potential of PTE to ameliorate HFD-induced obesity in vivo.

## 1. Introduction

Microalgae constitute a sustainable source of a multitude of nutrients with interesting properties such as proteins, ω-3 fatty acids, carotenoids, vitamins, and minerals [1], leading to an increasing market of microalgae-containing nutraceuticals and food products with important clinical and economic implications [2]. The marine diatom microalga *Phaeodactylum tricornutum* has potential for use in animal feed and human nutrition especially because it contains polyunsaturated fatty acids (PUFAs) and phytochemicals (e.g., polyphenols and carotenoids) [3,4]. *P. tricornutum* is particularly enriched in the ω-3 PUFA eicosapentanoic acid (EPA) [5,6] and the carotenoid fucoxanthin [6,7], which likely mediate the physiological and nutritional value of this microalga. Beneficial health effects such as anti-inflammatory [8,9,10,11,12], anti-obesity, and anti-diabetic effects [13,14,15,16,17,18] have been reported in cell and in vivo studies for these two compounds, mostly derived from fish oil (EPA) and edible macroalgae (fucoxanthin). The anti-obesity effects of ω-3 long-chain PUFA comprise decreased lipogenesis and the enhancement of fatty acid oxidation in liver and adipose tissues [13,19]. Fucoxanthin anti-obesity activity has been attributed to the stimulation of thermogenesis by increasing the expression of uncoupling protein 1 (UCP1) in adipose tissues [16,17,20] as well as to effects on intestinal lipid absorption and lipid metabolism [17,20,21,22,23]. UCP1 is a mitochondrial inner membrane protein, typically expressed in brown adipose tissue (BAT) and inducible in white adipose tissue (WAT) through a process known as WAT browning or beigeing [24], whose activity allows the dissipation of substrate-derived energy as heat.

Despite its interesting composition, few studies to date have addressed the anti-obesity properties of *P. tricornutum* in vivo [25,26]. In these studies, supplementation of the diet with *P. tricornutum* lipid extract [25] or *P. tricornutum* powder [26] ameliorated body weight and body fat gain of mice on a high fat diet (HFD) independently of decreases in food intake. In the study of Kang et al., supplementation was also shown to ameliorate HFD-induced metabolic derangements, such as hyperglycemia, hyperlipidemia, and insulin resistance, and to exert antioxidant effects in the liver [25]. In the study by Kim et al., evidence was provided that *P. tricornutum* powder may activate the AMP-activated protein kinase (AMPK) pathway in the liver [26]. However, these previous reports did not address changes in cellular and metabolic features of adipose tissues as potential contributors to the anti-obesity activity of *P. tricornutum* supplementation.

We here aimed to investigate the ability of a lipophilic ethanol extract of *P. tricornutum* (PTE) to oppose the development of obesity in obesity-prone (C57BL/6J) mice fed an obesogenic HFD, with focus on effects in adipose tissues. Therefore, body weight gain, adipose depots weight, adipocyte size distribution, and expression in adipose tissues of selected genes related to lipid and energy metabolism were analyzed, together with parameters related to glucose control.

## 2. Materials and Methods

### 2.1. Materials

Chemicals were purchased from Merck (Darmstadt, Germany), Sigma-Aldrich (Taufkirchen, Germany), and VWR (Bruchsal, Germany) or from Carl Roth (Karlsruhe, Germany), unless otherwise noted.

### 2.2. Microalgae Cultivation, Processing, and Preparation of Ethanolic Extract

The *P. tricornutum* strain UTEX 640 (SAG 1090-1b) was obtained from the culture collection of Algae (SAG) from the University of Goettingen (Germany) and was cultivated under controlled and axenic conditions, as described previously [27]. The biomass was harvested by centrifugation, the supernatant was discarded, and the remaining pellets were stored at −20 °C until cell disruption. The biomass of several cultivations was combined and lyophilized, and it was protected from light in a Christ Alpha 1–2 LD freeze drier (Osterode a. Harz, Germany). This was followed by cell disruption using the tissue homogenizer Precellys 24 from Bertin Technologies (Frankfurt/Main, Germany). The resulting *P. tricornutum* powder was applied to pressurized liquid extraction (ASE 350, Thermo-Fisher Scientific, Waltham, MA, USA) in accordance with the method described earlier by Derwenskus et al. using ethanol as extraction solvent [6]. The obtained extract was aliquoted, the ethanol was evaporated under a stream of nitrogen, and the extract was stored at −80 °C until used for animal experiments. In order to apply PTE to the mice, the dried extract was resolved in olive oil:water (2:1, *v*:*v*) to achieve a concentration of 0.1 mg/µL or 0.3 mg/µL and homogenized in an ultrasonic bath for 5 min.

### 2.3. Animal Experiment

The study was approved by the Bioethical Committee of the University of the Balearic Islands (UIB, Ref. CEEA 43/07/15). International standards for the use and care of laboratory animals were followed. C75Bl/6J mice were originally obtained from Charles River Laboratories (Barcelona, Spain) and expanded at the UIB animal house. The animals were housed in standard cages (without running wheel) at 22 °C with a 12-h light/dark cycle and ad libitum access to food (chow) and water. One week prior to the start of the experiment, six- to eight-week-old male C75BL/6J mice were divided into three groups with six animals per group (three animals per cage) and switched from chow to a defined low fat diet (3.8 kcal/g, 10% energy as fat, Research Diets D12450J, New Brunswick, NJ, USA). The diet was then changed to a defined HFD containing 4.7 kcal/g and 45% energy as fat (Research Diets D12451). At the same time as the HFD, the animals received daily, orally with the aid of a pipette, the vehicle (olive oil:water, 2:1, *v*:*v*) (placebo group) or PTE at a dose of 100 mg/kg body weight (bw)/day (PTE100) or 300 mg/kg bw/day (PTE300). Body weight and food intake were regularly monitored. Food intake was estimated on a per-cage basis, from the actual amount of food consumed by the animals and its caloric equivalence. Body composition was analyzed using an Echo MRI body composition analyzer (EchoMRI, LLC, Houston, TX, USA). At day 22, animals were starved for 6 h (from 06:00 a.m. to 12:00 p.m.) after which tail blood was collected for the measurement of circulating parameters. After 26 days, the animals were euthanized. Blood, liver, and adipose tissues (including interscapular BAT and epididymal, inguinal, and retroperitoneal WAT) were dissected and stored at −80 °C until used for analysis. Samples of the liver, BAT, and inguinal WAT were fixed for histology.

### 2.4. Circulating Parameters

Blood glucose was determined using an Accu-Chek Aviva system (Roche Diagnostics, Risch, Switzerland). Commercial kits for measurement of serum insulin (Mercodia, Uppsala, Sweden), non-esterified fatty acids (NEFA; Wako Chemicals GmbH, Neuss, Germany) and triacylglycerides (TAG; Sigma-Aldrich, St. Louis, MO, USA) were applied following the manufacturer’s protocols. The homeostatic model assessment for insulin resistance (HOMA-IR) and the revised quantitative insulin sensitivity check index (R-QUICKI) were calculated as described earlier [28].

### 2.5. Total Liver Fat Content

The total fat content in the liver was determined by Folch extraction with minor modifications [29]. In brief, 50–80 mg of fresh liver was weighted in a sample tube, followed by adding 500 µL of PBS and homogenization with a sonication probe for 10 s. To each sample, 500 µL methanol were added, mixed thoroughly for 2 min, followed by addition of 1 mL chloroform and mixing for 2 min. The mixture was centrifuged for 3 min at 4000× *g*, and the lower chloroform phase was transferred to a new tube using a glass pipette. The extraction procedure was repeated three times, and the solvent was evaporated under a stream of nitrogen. The tube with the dried residue was weighed, and the total fat content was calculated by subtracting the weight of the empty tube.

### 2.6. Carotenoid and Fatty Acid Analyses

Fatty acids in PTE were analyzed, as described earlier [6], using gas chromatography and a flame ionization detector. Carotenoids in PTE and in liver, BAT, and epididymal and inguinal WAT samples of animals were analyzed by HPLC coupled to a photodiode array detector, as described previously [6,30].

### 2.7. Total RNA Isolation and Quantitative Real Time PCR (qPCR) Analysis

Total RNA was extracted from tissues or cells using TRI Reagent (Sigma-Aldrich, St. Louis, MO, USA) following the manufacturer’s instructions. Isolated RNA was quantified using NanoDrop ND-1000 spectrophotometer (NanoDrop Technologies Inc., Wilmington, DE, USA) and its integrity confirmed by agarose gel electrophoresis. A 0.25 µg sample of total RNA was reverse-transcribed using reagents from Life Technologies (Carlsbad, CA, USA). The resulting cDNA was subjected to qPCR analysis on a StepOnePlus instrument (Life technologies). *Hprt-1* transcript was used as a reference housekeeping gene. The sequences of the employed primers for qPCR are available on request.

### 2.8. Histology and Immunohistochemistry

Tissue samples were fixed by immersion in 4% paraformaldehyde in 0.1 M sodium phosphate buffer, pH 7.4, overnight at 4 °C, dehydrated in a graded series of ethanol, cleared in xylene, and embedded in paraffin blocks for light microscopy. Five-micrometer-thick sections of tissues were cut with a microtome, mounted on slides, and stained with hematoxylin/eosin. Morphometric analysis of inguinal WAT sections was performed by digital acquisition of adipose tissue areas using AxioVision 40V 4.6.3.0 software and a Zeiss Axioskop 2 microscope equipped with an AxioCam ICc3 digital camera (Carl Zeiss S.A., Barcelona, Spain). Distributions of adipocyte size were obtained from individual data of cell sizes. Immunohistochemical detection of UCP1, mitofusin-2 (MFN2), and galectin-3 in fixed tissue sections was performed, essentially as previously described [31], using polyclonal antibodies against UCP1 (Catalog number GTX112784, GeneTex, Irvine, CA, USA), Mfn2 (Catalog number HPA030554, Sigma-Aldrich, St. Louis, MO, USA), and galectin-3 (MAC-2; Catalog number CL8942AP, Cedarlane, Burlington, Ontario, Canada).

### 2.9. Immunoblotting

Total protein was isolated from tissues using TRI Reagent (Sigma-Aldrich, St. Louis, MO, USA) following the manufacturer’s instructions. Protein concentration was determined with Pierce^TM^ BCA Protein Quantification Assay kit (Thermo-Fisher Scientific, Waltham, MA, USA). Ten micrograms of protein was loaded and separated in a precast 12% gel (Bio-Rad, Hercules, CA, USA) and transferred onto a 0.2 µm nitrocellulose membrane using a Trans-Blot Turbo semi-dry transfer apparatus (Bio-Rad, Hercules, CA, USA). Membranes were blocked for 1 h at room temperature with Odyssey Blocking Buffer (Li-Cor, Lincoln, NE, USA) and incubated overnight at 4 °C with gentle shaking with primary antibodies (1:1000 in Tris Buffered Saline-Tween 20, TBS-T) against UCP1 and MFN2 (same sources as in 2.8). Membranes were then incubated with the corresponding secondary IRDye antibodies (1:10000 in TBS-T, 1 h at room temperature), and the signal was detected using an Odyssey near-infrared scanner (Li-Cor, Lincoln, NE, USA).

### 2.10. Cell Culture Experiment

3T3-L1 preadipocytes obtained from ATCC (American Type Culture Collection, Manassas, VA, USA) were grown and differentiated in six-well culture plates, using commercial media from Zen-Bio Inc (Research Triangle Park, NC, USA) and following the manufacturer standard protocol. Preadipocytes were routinely cultured in Preadipocyte medium at 37 °C and 5% CO_2_. For differentiation into adipocytes, the cells were allowed to reach confluence, and two days later (defined as day zero) the preadipocyte medium was replaced by Differentiation medium. On day three, differentiation medium was replaced by Adipocyte Maintenance medium. Cells were cultured until day eight, with medium replacement every two days. Adipogenic differentiation of the cells was regularly monitored through phase contrast microscopical examination. On day seven, when more than 95% of the cells showed intracellular lipid accumulation, cells were treated with PTE (100 mg/L), fucoxanthin (5 µM; Sigma-Aldrich, St. Louis, MO, USA) or vehicle (ethanol; 10 µL) for 24 h prior to harvesting. In parallel plates intracellular lipid content was quantified by Oil Red O (ORO) staining, as previously described [32], evidencing no differences between treatments (results not shown). The treatments applied had no cytotoxic effects in 3T3-L1 preadipocytes as assessed by the lactate dehydrogenase release assay (results not shown).

### 2.11. Statistical Analysis

Data are presented as mean ± standard deviation (SD) or as mean ± standard error of the mean (SEM). Comparisons between three groups (placebo, PTE100, and PTE300) were assessed by a non-parametric Kruskal–Wallis test. To compare between two groups, the non-parametric Mann–Whitney U test was used. In both cases, threshold of significance was set at *p* < 0.05. IBM SPSS Statistics for Windows, version 23.0 (IBM Corp., Armonk, NY, USA) was used for the analyses.

## 3. Results

### 3.1. Phaeodactylum tricornutum Extract (PTE) Characterization

The extract contained 15 mg dry matter (dm)/mL. Table 1 shows the fatty acid and carotenoid composition of PTE. Palmitoleic acid, palmitic acid, and EPA represented the main fatty acids. The PTE contained relatively high amounts of the carotenoid fucoxanthin and lower amounts of zeaxanthin and β-carotene.

### 3.2. PTE Supplementation Led to an Accumulation of Fucoxanthin Metabolites in Adipose Tissues

Animals received through PTE supplementation a daily dose of ~2.4 mg fucoxanthin/kg bw (PTE100) or ~7.1 mg fucoxanthin/kg bw (PTE300). Fucoxanthin is rapidly metabolized to fucoxanthinol through deacetylation during intestinal digestion, so that little is absorbed intact, and fucoxanthinol is further dehydrogenated/isomerized to amarouciaxanthin A in the liver and other tissues [33,34,35]. Traces of fucoxanthin metabolites, potentially the sum of fucoxanthinol and amarouciaxanthin A [30], were found in the interscapular BAT of PTE300-supplemented mice and in the epididymal and inguinal WAT depots of PTE100-supplemented mice, whereas supplementation with PTE300 resulted in a consistent accumulation up to 1.33 ± 0.72 µg fucoxanthinol/g epididymal WAT and 1.48 ± 0.76 µg fucoxanthinol/g inguinal WAT. Levels of fucoxanthin metabolites in the liver of all experimental groups and the adipose tissues of the control (HFD-fed, placebo-treated) group were below detection. Lack of detection of fucoxanthin metabolites in the liver of PTE-supplemented mice might be consistent with previous findings that fucoxanthin metabolites have a shorter half-life in the liver than in adipose tissues [36].

### 3.3. PTE Supplementation Partly Counteracted High Fat Diet (HFD)-Induced Obesity

**Figure 1:**
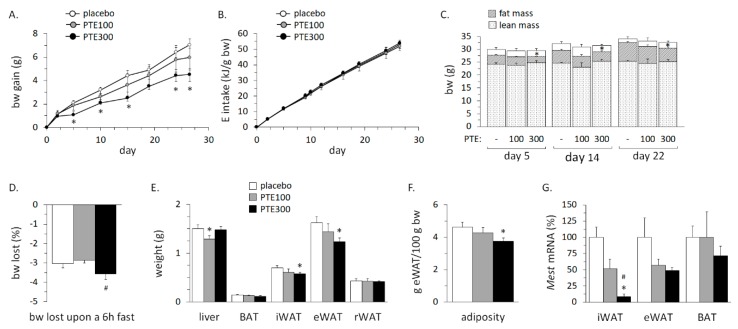
*Phaeodactylum tricornutum* ethanolic extract (PTE) ameliorates fat deposition in C57BL/6J mice fed with a high fat diet (HFD). Evolution of body weight (bw) gain (**A**), cumulative energy intake from food (**B**), and body composition (**C**) from day 1 to 26 of dietary challenge. Body weight lost upon a 6 h fast, on day 22 (**D**). Liver, interscapular brown adipose tissue (BAT), and inguinal, epididymal, and retroperitoneal white adipose tissue (iWAT, eWAT, and rWAT) weights (**E**), adiposity as eWAT weight as percent body weight (**F**), and mRNA expression levels of *Mest* in iWAT, eWAT, and BAT (**G**) at the end of the experiment. HFD-fed mice received daily an oral dose of PTE (100 mg or 300 mg/kg bw) or placebo (olive oil:water, 2:1, *v*:*v*) for 26 days. Data are mean ± SEM of 5–6 male mice/group. To compare between two groups, the non-parametric Mann–Whitney U test was used: *, different (*p* < 0.05) from vehicle; and #, different (*p* < 0.05) between doses.

In general, PTE supplementation was well accepted by the animals, and there were no apparent effects on spontaneous physical activity or adverse health effects. The average initial body weight was 27.7 ± 0.5 g and did not differ significantly among the three experimental groups. HFD feeding led to a gradual increase of body weight in the three groups, which was already evident after two days. The PTE-treated mice gained less body weight than controls upon HFD feeding, an effect that reached statistical significance for PTE300 (Figure 1A). At the end of the experiment (day 26), control, PTE100, and PTE300 mice had gained 7.0 ± 0.5, 6.0 ± 1.0, and 4.5 ± 0.5 g; final body weights were 35.1 ± 0.6, 33.5 ± 1.4, and 33 ± 0.7 g, respectively. Differences in body weight gain were not because of the differences in energy intake, which was similar in the three experimental groups throughout the entire duration of the HFD challenge (Figure 1B). The PTE300 group also revealed less total body fat mass than controls in body composition analyses performed after 5, 14, and 22 days on the HFD (Figure 1C). Body weight lost upon a 6 h fast, used as an indicator of energy expenditure, was maximal in the PTE300 group (Figure 1D). In keeping with these results, at the end of the study the mass of the epididymal and inguinal WAT depots was significantly lower (by 24% and 17%, respectively) in the PTE300 group compared with the control group (Figure 1E). There were no differences between groups in retroperitoneal WAT mass and BAT mass (Figure 1E). The weight of the epididymal depot expressed as percentage of body weight, which is commonly used as an adiposity index in mice [37], was significantly lower in the PTE300 group compared to the control group (Figure 1F). Furthermore, mRNA expression levels of mesoderm-specific transcript homolog protein (*Mest*), used as a marker of WAT expansion [38,39], were markedly decreased in inguinal WAT of PTE-supplemented mice (Figure 1G). Histological analysis of the liver was largely normal and did not reveal obvious hepatosteatosis in any of the experimental groups, possibly because of the relatively short period of HFD feeding applied (Supplementary Figure S1A). Biochemical analysis showed a tendency for lower total liver lipid content in the PTE-supplemented mice (Supplementary Figure S1B).

**Figure 2:**
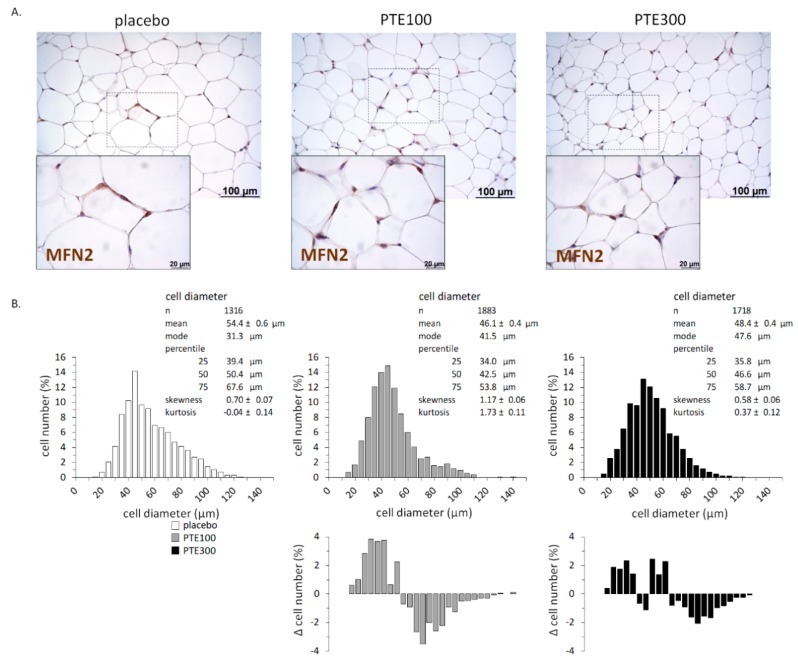
*Phaeodactylum tricornutum* ethanolic extract (PTE) decreases adipocyte size in inguinal white adipose tissue (iWAT) of C57BL/6J mice fed with a high fat diet (HFD). Representative microphotographs illustrating adipocyte size and Mitofusin (MFN) 2 immunostaining (**A**), and distribution of adipocytes size (**B**) in iWAT at the end of the experiment. HFD-fed mice received daily an oral dose of PTE (100 mg or 300 mg/kg body weight) or placebo (olive oil:water, 2:1, *v*:*v*) for 26 days. Five to six animals per group and between 200 and 300 cells per animal were included in the analysis of distribution of adipocytes size. The area of individual adipocytes was measured using a quantitative morphometric method at 20× magnification with the assistance of Axio Vision software. Adipocyte size distribution was statistically different (*p* < 0.001) between the control and the PTE groups, according to the Kolmogorov–Smirnov test. The bottom panels in (**B**) correspond to the difference in frequency for each adipocyte size interval between the PTE-supplemented group (PTE100 or PTE300) and the control (vehicle receiving) group.

Microscopical examination evidenced that inguinal WAT adipocytes were smaller in PTE-supplemented mice than in control mice (see representative microphotographs in Figure 2A). Detailed morphometric analysis confirmed a shift of adipocyte population distribution toward an increased percentage of small adipocytes and a lower percentage of large adipocytes in PTE-supplemented mice compared to controls (Figure 2B). The Kolmogorov–Smirnov test indicated that the difference in distributions of cell size between control and PTE-supplemented mice was statistically significant (*p* < 0.001 for both PTE doses) (Figure 2B). Microscopical examination of inguinal WAT sections also revealed a sporadic occurrence of crown-like structures (CLSs) in two out of five control mice and three out of five PTE100 mice examined, and an even more consistent occurrence of CLS was found in the PTE300-supplemented group, for which CLSs were detected in all five animals examined (Supplementary Figure S2). These CLSs were positive for immunostaining against the macrophage marker galectin-3 (MAC-2).

Table 2 shows parameters related to glucose control and insulin sensitivity determined after a short fasting on day 22 of HFD challenge. There was a tendency for decreased HOMA-IR index (*p* = 0.068; Mann–Whitney U test) and increased R-QUICKI index (*p* = 0.100; Mann–Whitney U test) in the PTE100-supplemented group, due to lower levels of fasting glucose as well as (though non-significantly) insulin in blood of animals in this group compared to controls. These results suggested improved glucose control and insulin sensitivity on HFD in the PTE100 mice. Such trends were absent in the PTE300-supplemented mice, which had fasting blood glucose levels significantly higher than control and PTE100-supplemented mice, and fasting insulin, NEFA, HOMA-IR, and R-QUICKI indexes were very similar to those of control mice. Fed blood glucose levels at the end of the experiment did not significantly differ between groups (control, 180 ± 8.1; PTE100, 186 ± 9.8; PTE300, 191 ± 10.7 mg/dL).

### 3.4. PTE Supplementation Affected Transcriptional Control of Lipid Metabolism in White Adipose Tissue (WAT) Depots and Favored Browning of Subcutaneous WAT

**Figure 3:**
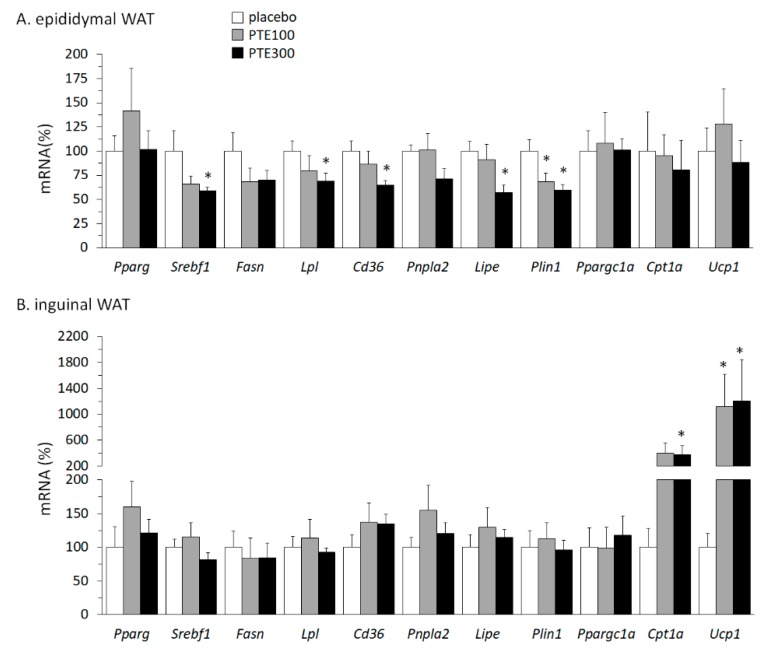
*Phaeodactylum tricornutum* ethanolic extract (PTE) down-regulates fatty acid uptake and lipid turnover capacities in epididymal white adipose tissue (eWAT) and increases oxidative/thermogenic capacity in inguinal WAT (iWAT) of C57BL/6J mice fed with a high fat diet (HFD). mRNA levels of selected genes as indicated were analyzed in eWAT (**A**) and iWAT (**B**) at the end of the experiment. HFD-fed mice received daily an oral dose of PTE (100 mg or 300mg/kg body weight) or placebo (olive oil:water, 2:1, *v*:*v*) for 26 days. Data are the mean ± SEM of 5–6 male mice/group and are expressed relative to the mean value of the vehicle group, which was set to 100. To compare between two groups, the non-parametric Mann–Whitney U test was used: *, different (*p* < 0.05) from vehicle.

Gene expression of key proteins related to different aspects of fatty acid and energy metabolism was compared in visceral (epididymal) and subcutaneous (inguinal) WAT depots of control and PTE-supplemented mice under HFD. In the epididymal WAT of PTE-supplemented mice, mRNA levels of lipolysis-related genes *Lipe*, encoding hormone sensitive lipase, and *Plin1*, encoding perilipin 1, were significantly down-regulated (*p* = 0.039 and *p* = 0.033, respectively; Kruskal–Wallis test), and there were trends to down-regulation for the lipogenesis-related gene *Srebf1* and the fatty acid uptake-related gene *Cd36* as well (*p* = 0.099 and *p* = 0.078, respectively; Kruskal–Wallis test) (Figure 3A). Further, mRNA levels in the epididymal fat depot of *Lpl*, coding for lipoprotein lipase that enables utilization of fatty acids from circulating triacylglycerols, were significantly down-regulated in the PTE300 mice relative to controls.

In subcutaneous (inguinal) WAT, expression of these same genes was unaffected by PTE supplementation (Figure 3B). However, *Cpt1* was 4 times and *Ucp1* at least 11 times upregulated in inguinal WAT of PTE-supplemented mice, indicating an increased capacity for fatty acid oxidation and thermogenesis (Figure 3B) (*p* = 0.085 and *p* = 0.022, respectively; Kruskal–Wallis test). Further, at the protein level, UCP1 could not be detected in inguinal WAT in any control mice, but it was detected by immunoblotting in one-sixth PTE100 and two-fifths PTE300 mice analyzed (results not shown). Moreover, immunostaining of inguinal WAT sections for MFN2—an outer mitochondrial membrane protein whose activity has been linked to an enhancement of oxygen consumption and substrate oxidation [40]—was more intense in PTE-supplemented mice than in controls (see the brown color in the periphery of adipocytes in Figure 2A).

### 3.5. PTE Supplementation Favored Brown Adipose Tissue (BAT) Activation

**Figure 4:**
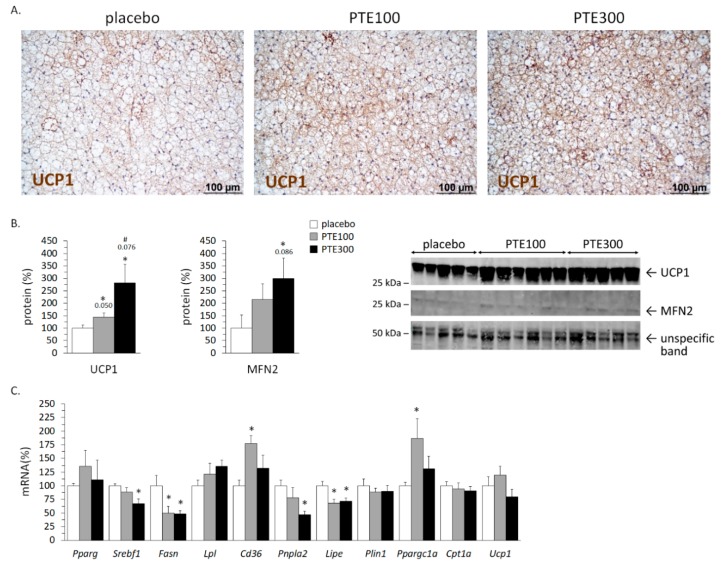
*Phaeodactylum tricornutum* ethanolic extract (PTE) activates interscapular brown adipose tissue (BAT) in C57BL/6J mice fed with a high fat diet (HFD). Representative microphotographs illustrating BAT activation and Uncoupling protein (UCP) 1 immunostaining (**A**), UCP1 and Mitofusin (MFN) 2 protein levels as determined by immunoblotting in BAT (**B**), and mRNA levels of selected genes in BAT (**C**) at the end of the experiment. HFD-fed mice received daily an oral dose of PTE (100 mg or 300 mg/kg body weight) or placebo (olive oil:water, 2:1, *v*:*v*) for 26 days. Data are the mean ± SEM of 5–6 male mice/group and are expressed relative to the mean value of the vehicle group, which was set to 100. To compare between two groups the non-parametric Mann–Whitney U test was used: *, different from vehicle; and #, different between doses. Threshold of statistical significance was set at *p* < 0.05; in (**B**), *p* values < 0.1 are also indicated.

PTE supplementation led to BAT activation as indicated by the smaller size of brown adipocytes and their enrichment in UCP1 protein immunostaining (see the representative microphotographs in Figure 4A). This was confirmed by immunoblotting analysis of UCP1 and MFN2, showing dose-dependent increased levels of both proteins in BAT of PTE-supplemented animals as compared to controls (Figure 4B). Moreover, PTE-supplemented mice showed an increased gene expression in BAT of *Cd36* (*p* = 0.046*;* Kruskal–Wallis test) that was especially evident in the PTE100 group, which also showed increased mRNA levels of *Ppargc1a* in BAT (Figure 4C). PTE supplementation had no effect on the expression of *Ucp*1 or *Cpt1* at the mRNA level, and resulted in a downregulated expression in BAT of the lipolytic genes *Lipe* and *Pnpla2* (*p* = 0.029 and *p* = 0.057, respectively; Kruskal–Wallis test), encoding hormone sensitive lipase and adipose triglyceride lipase. mRNA levels of the lipogenic genes *Fasn* and *Srebf1* were also down-regulated in BAT of PTE-supplemented mice (*p* = 0.049 and *p* = 0.074, respectively; Kruskal–Wallis test) (Figure 4C).

### 3.6. PTE and Fucoxanthin had Both Overlapping and Distinct Effects on Gene Expression of Lipid Metabolism-Related Genes in Mature 3T3-L1 Adipocytes

**Figure 5:**
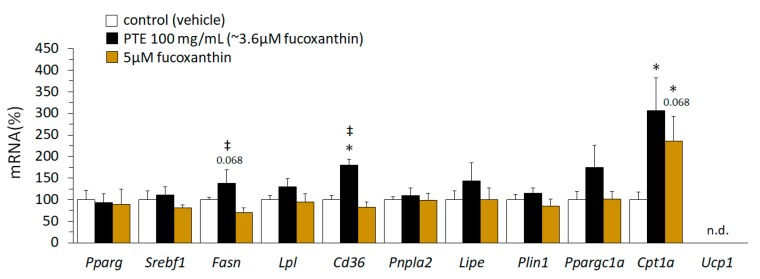
*Phaeodactylum tricornutum* ethanolic extract (PTE) and fucoxanthin effects on gene expression in mature 3T3-L1 adipocytes are not equivalent. mRNA levels of selected genes in mature 3T3-L1 adipocytes are shown. 3T3-L1 preadipocytes were grown and differentiated following a standard protocol. On day 7, cultures were treated with PTE (100 mg/L), fucoxanthin (5 µM; Sigma-Aldrich), or vehicle (ethanol 0.5%) for 24 h. Data are the mean ± SEM of two independent experiments made in triplicate and are expressed relative to the mean value of the vehicle group, which was set to 100. To compare between two groups the non-parametric Mann–Whitney test was used: *, different from vehicle; and ‡, different from fucoxanthin. Threshold of statistical significance was set at *p* < 0.05, *p* values < 0.1 are also indicated.

Expression levels of a series of genes related to lipid metabolism and thermogenesis were compared in mature 3T3-L1 adipocytes exposed to the vehicle (control cells) or to either 100 mg PTE/L, contributing ~3.6 μM fucoxanthin, or a similar dose of pure fucoxanthin (5 μM) for 24 h (Figure 5). *Cpt1a* mRNA levels were strongly, relative to levels in control cells, similarly induced by both PTE and fucoxanthin exposure. *Cd36* mRNA levels were induced following exposure to PTE, but not fucoxanthin, whereas *Fasn* mRNA levels were decreased following exposure to fucoxanthin, but not PTE. *Ucp1* mRNA could not be detected in mature 3T3-L1 adipocytes irrespective of treatment.

## 4. Discussion

The current obesity pandemic [41] is boosting research on the use of plant and algae-based products including extracts or isolated components in obesity prevention and therapy [42,43]. Adipose tissues are active players in energy metabolism and a target of anti-obesity strategies. WAT is the main storage site of excess energy taken up from food, and both WAT and BAT are plastic tissues where substrate (mainly fatty acid) oxidation and thermogenesis can be activated, through pharma or food compounds, to oppose body fat accrual and preserve metabolic health [44,45,46,47]. In this work, we provide evidence that an ethanolic extract of the microalga *Phaeodactylum tricornutum* ameliorates the development of diet-induced obesity and insulin resistance in mice independent of decreased food intake. *P. tricornutum* supplementation was linked to increased energy expenditure, as indicated by increased body weight loss upon fasting, molecular and histological signs of BAT activation, and molecular signs of enhanced mitochondrial oxidative metabolism in subcutaneous WAT. Thus, the results point to thermogenesis and metabolic activation in adipose tissues as one mechanism for the anti-obesity effects of PTE, even if (as a limitation of the study) indirect calorimetry measurements of energy expenditure are lacking. Other mechanisms that could be involved are decreased dietary lipid absorption, since energy excreted in feces was not measured, and increased spontaneous physical activity, since this parameter was not continuously monitored.

An effect of lipophilic constituents of *P. tricornutum* on obesity-related metabolic changes was suggested by previous findings. In particular, studies have reported anti-obesity effects of isolated fucoxanthin or fucoxanthin-containing extracts of edible seaweeds (macroalgae) such as *Undaria pinnatifida* or *Laminaria japonica* in genetic and dietary rodent models of obesity, which have been ascribed to metabolic effects in tissues including, notably, the adipose tissues [16,17,20,23]. *P. tricornutum* is 10 times richer in fucoxanthin than macroalgae [7]. Further, in previous studies we found a dose-dependent accumulation of fucoxanthin metabolites in adipose tissues of mice that were fed diets containing 5% to 25% *P. tricornutum* biomass [30,48]. This scenario prompted us to assay the anti-obesity activity of an ethanolic extract of *P. tricornutum* (PTE) with focus on its impact on energy and lipid metabolism in white and brown adipose tissues.

Mice on HFD receiving PTE supplementation accumulated fucoxanthin metabolites in adipose tissues, as expected [30,48], and displayed lower body weight gain, body fat content, and weight of WAT depots than control mice receiving placebo. Whereas effects on macroscopic biometric parameters were observed mainly at the high PTE300 dose, it is noteworthy that favorable effects of supplementation on adipocyte size and distribution (e.g., decreased mean adipocyte size, increased proportion of small adipocytes, and decreased proportion of large adipocytes) were already evident in inguinal WAT at the low PTE100 dose. Smaller adipocytes in obesity have been linked to a better metabolic profile, both in humans [49] and rodent models [50]. Decreased *Mest* mRNA levels found in WAT depots of PTE-supplemented mice are also in keeping with PTE opposing the development of obesity, since *Mest* expression is a known predictive marker of WAT expansion sensitive to dietary anti-obesity interventions [38,39]. Two previous papers evidencing anti-obesity effects of *P. tricornutum* employed high or very high doses as lipid extract (0.7% in diet, corresponding to ~800 mg extract/kg bw per day or ~255 mg fucoxanthin/kg bw per day) [25] or dry powder (15% and 30% in diet, corresponding to ~14 and 36 g dry powder/kg bw per day or ~72 and 155 mg fucoxanthin/kg bw per day) [26], which were well over the doses used in the present work (100 and 300 mg extract/kg bw per day or 2.4 and 7.1 mg fucoxanthin/kg bw per day). Further, these previous studies did not address cellular or molecular effects of supplementation on adipose tissues.

Both visceral (epididymal) and subcutaneous (inguinal) WAT were reduced in mass in the PTE–supplemented animals compared to controls on the HFD, and molecular results suggest that metabolic effects in both depots may contribute to the local depot weight reduction and the whole-body anti-obesity effect of supplementation. On the one hand, gene expression data are suggestive of decreased lipid uptake and turnover in the visceral (epididymal) fat depot of PTE-supplemented mice, where gene expressions related to fatty acid uptake (*Lpl* and *Cd36*), fatty acid mobilization from intracellular lipid stores (*Pnpla2*, *Lipe*, and *Plin1*), and fatty acid and triacylglycerol synthesis (*Srebf1* and *Fasn*) were simultaneously found to be down-regulated. A decreased lipid turnover in visceral fat might be of interest in the context of obesity, as it has been suggested that increased lipid turnover in visceral WAT (and/or decreased turnover in subcutaneous WAT) may result in metabolic complications of overweight or obesity [51].

On the other hand, results herein point to an effect of PTE supplementation favoring the browning of subcutaneous (inguinal) WAT. Thus, although we lacked to detect the appearance of adipocytes with the typical brown adipocyte multilocular distribution of intracellular fat, molecular signs of an enhanced capacity for oxidative metabolism and thermogenesis were present in the inguinal WAT of PTE-supplemented mice. These included an increased expression of *Ucp1* (and probably UCP1), which is the hallmark of WAT browning, and also *Cpt1a* and MFN2. The *Cpt1a* protein product is traditionally considered the rate-limiting enzyme for long-chain fatty acid uptake and beta-oxidation by mitochondria [52]. MFN2 is a protein involved in mitochondria dynamics that favors mitochondria fusion and whose activity enhances mitochondrial oxidative metabolism in cells [40].

Not only WAT browning but also increased phagocytosis of large adipocytes could contribute to the decreased inguinal WAT mass and the favorable changes in adipocyte size distribution observed in PTE-supplemented mice relative to controls on the HFD. This is suggested by the increased occurrence of CLS in WAT of PTE-supplemented mice, especially at the high PTE300 dose. CLS corresponds to dead adipocytes that are being cleared by surrounding macrophages [53]. The biological significance of these structures is not straightforward. Massive macrophage infiltration in WAT (especially visceral WAT) in obesity has been related to pro-inflammatory cytokine production, chronic inflammation, and systemic insulin resistance [54], yet there is emerging evidence for beneficial functions of WAT macrophages during diet-induced obesity, as the clearance of dead adipocytes may promote adipocyte turnover [55,56]. Further studies are required to discard a pro-inflammatory potential of PTE, yet we note that the increased presence of CLS in inguinal WAT of PTE-supplemented mice did not associate with an aggravation of insulin resistance on the HFD. On the contrary, PTE100 supplementation resulted in lower fasting blood glucose levels—in keeping with reported antihyperglycemic effects of fucoxanthin [12]—and the insulin resistance index (HOMA-IR) of PTE-supplemented mice was tendentially lower (PTE100) or indistinguishable (PTE300) from that of control mice. Further, anti-inflammatory effects have been reported for *P. tricornutum* extracts in cell studies in human blood mononuclear cells and murine macrophages [57] and for fucoxanthin metabolites in adipocytes [12,58], and fucoxanthin supplementation is shown to improve skeletal muscle insulin responsiveness in mice with genetic diabesity [59]. However, our results do suggest that lower doses of PTE might exert better effects than higher doses on glucose control and insulin sensitivity.

Observed effects in BAT most likely contribute to the ability of PTE supplementation to counteract the development of diet-induced obesity. Activation of BAT in the supplemented mice was very clear from the tissue microphotographs and the results of both BAT UCP1 immunohistochemical staining and immunoblotting. Activation of BAT was also consistent with the observed up-regulation of other genes and proteins known to be required for BAT thermogenesis, namely MFN2, *Cd36*, and *Ppargc1a*. MFN2 expression is shown to play a major role in BAT metabolism by physically coupling the mitochondria with lipid droplets and maintaining mitochondrial oxidative capacity [60]. *Cd36* encodes a transport protein present at both the plasma and the mitochondrial membrane that mediates fatty acid uptake in the cell and the mitochondria, and it is known to play an essential role in BAT thermogenesis [61,62]. *Ppargc1a* encodes PGC1α, a transcriptional coactivator first identified for its stimulatory role of BAT thermogenesis [63]. To be highlighted is the fact that PTE supplementation exerted opposite effects in visceral WAT and BAT regarding gene expression of proteins for cellular fatty acid provision and uptake (*Lpl* and *Cd36*), decreasing it in the epididymal depot and increasing it in BAT, while having no effects on these genes in the subcutaneous WAT depot. Overall, these results suggest that PTE supplementation in the context of HFD may favor channeling of dietary fatty acids away from visceral fat depots and toward ignition in thermogenic fat tissues, mainly BAT and also subcutaneous WAT.

In previous animal studies, supplementation with fucoxanthin or fucoxanthin-rich seaweed extracts led to inconsistent results regarding UCP1 expression in adipose tissues. Different authors observed either up-regulation [20] or lack of effect on UCP1 expression in BAT [16,64], yet in the latter reports BAT mass normalized to body weight increased following supplementation (contrary to WAT mass, which was decreased). Therefore, a contribution of increased BAT activity to observed anti-obesity effects cannot be discarded. An induction of UCP1 expression in visceral (gonadal) WAT following fucoxanthin supplementation has been reported and highlighted [16,17,20]; however, other reports failed to detect UCP1 induction in WAT of supplemented animals [64,65]. Further, visceral WAT has a minor tendency to turn to a BAT-like phenotype than subcutaneous (inguinal) WAT [66], yet most previous reports did not compare UCP1 induction in visceral and subcutaneous fat depots. As an exception, Wu et al. assessed gene expression related to mitochondriogenesis and thermogenesis in inguinal WAT, gonadal (epididymal) WAT, and BAT of mice fed obesogenic diets supplemented or not with fucoxanthin [64]. They confirmed an increase in the animals’ metabolic rate following fucoxanthin supplementation, and—different from our results in PTE-supplemented mice—they found little evidence of up-regulation of thermogenic genes in BAT and a similar up-regulation of many of such genes in visceral and subcutaneous WAT (though results for *Ucp1* did not reach statistical significance) [64].

Overall, while most animal studies to date point to anti-obesity properties of fucoxanthin, it would appear that parameters such as the time length of supplementation and the formulation of the fucoxanthin source (isolated vs. extract) influence the exact regulatory and metabolic mechanisms involved. In fact, effects of PTE and purified fucoxanthin on gene expression in mature 3T3-L1 adipocytes were not fully equivalent, suggesting that components other than fucoxanthin contribute to PTE effects in cultured adipocytes and likely also in supplemented animals in vivo. For different components of PTE including fucoxanthin, metabolic effects have been related to the induction of the PGC1α network in adipose tissues [64,67] and the activation of AMPK in tissues such as liver and muscle [68]. These two molecules are important players in the control of energy metabolism. While further mechanistic studies are warranted, we note that BAT activation brought about by PTE supplementation involved the induction of the PGC1α gene (Figure 4), but it did not affect levels of phosphorylated (active) AMPK nor the ratio phosphoAMPK/totalAMPK in the tissue (results not shown).

## 5. Conclusions

In summary, this work demonstrates that a *Phaeodactylum tricornutum* extract ameliorates the development of diet-induced obesity in a well-established rodent model, and it links this effect to the stimulation of oxidative metabolism in BAT and WAT depots, notably the induction of BAT recruitment and the browning of subcutaneous WAT. Knowledge on the anti-obesity action of *P. tricornutum* and its mechanisms may pave the way for novel uses of this microalga in the functional food and nutraceutical arena.

## Figures and Tables

**Table 1:** Fatty acid and carotenoid spectra of the ethanolic *Phaeodactylum tricornutum* extract (PTE).

Constituent	Concentration (µg/mg dm)
Fatty acids:	
Myristic acid	1.82 ± 0.10
Myristoleic acid	0.30 ± 0.02
Palmitic acid	7.73 ± 0.74
Palmitoleic acid	15.24 ± 0.44
cis-Oleic acid	1.52 ± 0.06
trans-Oleic acid	1.02 ± 0.05
α-Linoleic acid	0.71 ± 0.04
γ-Linoleic acid	0.30 ± 0.01
Eicosatrinoic acid	1.25 ± 0.05
Eicosapentanoic acid	7.32 ± 0.40
Carotenoids:	
Fucoxanthin	23.54 ± 0.60
Zeaxanthin	0.30 ± 0.05
β-Carotene	0.12 ± 0.04

dm—dry matter. Data are mean ± SD of three independent measurements.

**Table 2:** Plasma analyses and insulin resistance/sensitivity indexes in animals.

	Placebo	PTE100	PTE300
Glucose (mg/dL)	161 ± 8.2	134 ± 5.8 *	179 ± 5.5 *#
Insulin (mU/L)	42.1 ± 7.8	27.7 ± 1.9	33.5 ± 2.4
NEFA (mEq/L)	0.556 ± 0.046	0.714 ± 0.083	0.661 ± 0.113
HOMA-IR	17.0 ± 3.5	9.1 ± 0.71	14.7 ± 0.94 #
R-QUICKI	0.283 ± 0.005	0.295 ± 0.005	0.281 ± 0.005

Data are mean ± SEM; n = 5–6; Parameters were obtained from blood collected at day 22 after a 6 h fast. To compare between two groups, the non-parametric Mann–Whitney U test was used: *, different (*p* < 0.05) from vehicle; and #, different (*p* < 0.05) between doses; NEFA—non esterified fatty acids; HOMA-IR—homeostatic model assessment for insulin resistance; R-QUICKI—revised quantitative insulin sensitivity check index; PTE100—100 mg PTE/kg body weight/day; and PTE300—300 mg PTE/kg body weight/day.

## Supplementary Materials

The following are available online at https://www.mdpi.com/2072-6643/11/4/796/s1, Figure S1: Effects of PTE supplementation to C57BL/6J mice fed with a high fat diet on liver histological appearance and lipid content, Figure S2: Effects of PTE supplementation to C57BL/6J mice fed with a high fat diet on the appearance of crown-like structures in inguinal white adipose tissue.
