# Supplementary material for: A Lipophilic Fucoxanthin-Rich Phaeodactylum tricornutum Extract Ameliorates Effects of Diet-Induced Obesity in C57BL/6J Mice
Source: Nutrients. 2019 Apr 6;11(4):796. doi: 10.3390/nu11040796 (PMC6521120; doi:10.3390/nu11040796)
Supplement: Supplementary file 1 [file nutrients-11-00796-s001.docx]

Supplementary Materials of the article **A lipophilic fucoxanthin-rich *Phaeodactylum tricornutum* extract ameliorates effects of diet-induced obesity in C57BL/6J mice** by Andrea Gille, et al.


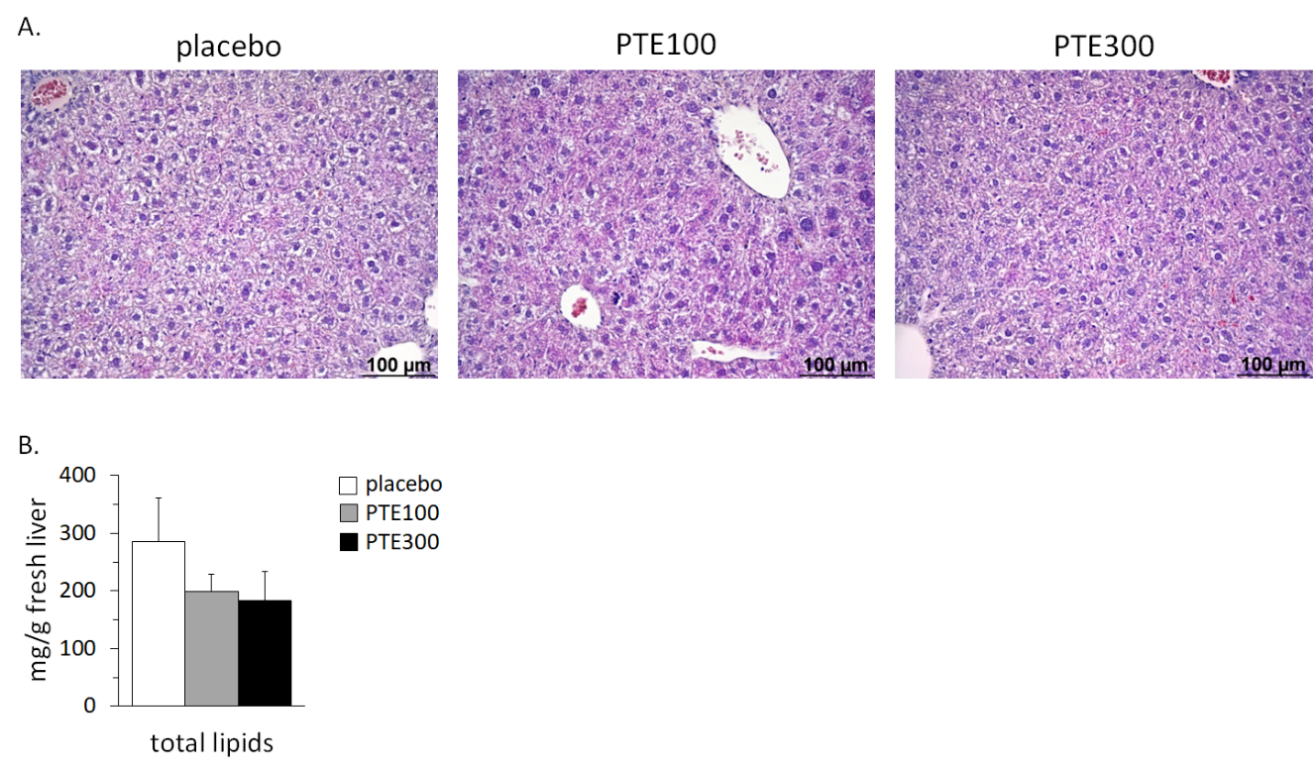


**Supplementary Figure S1.** *Phaeodactylum tricornutum* ethanolic extract (PTE) supplementation associated with a tendency to decreased lipid content in liver of C57BL/6J mice fed with a high fat diet (HFD). Representative microphotographs showing liver morphology (**A**), and hepatic lipid content (**B**) at the end of the experiment. HFD-fed mice received daily an oral dose of PTE (100 mg or 300mg/kg body weight) or placebo (olive oil:water, 2:1, v:v) for 26 days. Data are the mean±SEM of 5–6 male mice/group.

**
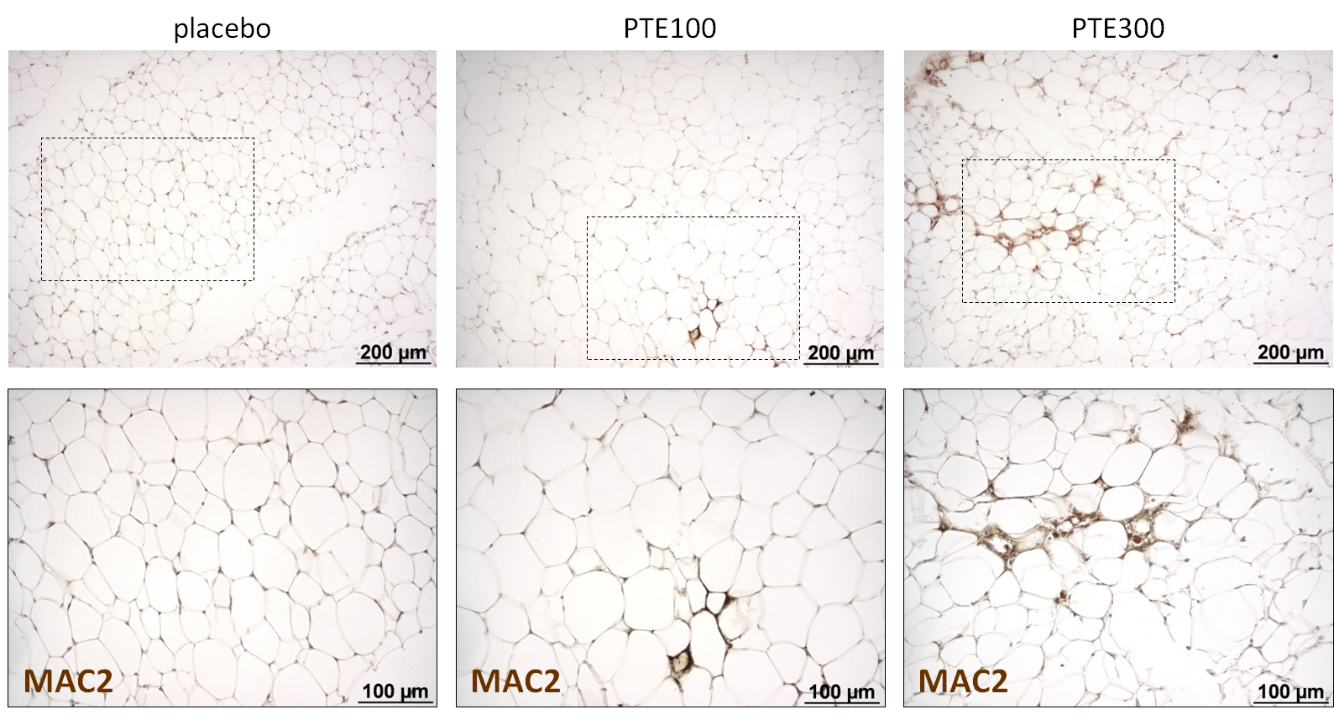
**

**Supplementary Figure S2.** *Phaeodactylum tricornutum* ethanolic extract (PTE) increases the presence of crown-like structures in inguinal white adipose tissue (iWAT) of C57BL/6J mice fed with a high fat diet (HFD). Representative microphotographs illustrating crown-like structures in iWAT at the end of the experiment, staining positive for galectin-3 protein (MAC2). HFD-fed mice received daily an oral dose of PTE (100 mg or 300mg/kg body weight) or placebo (olive oil:water, 2:1, v:v) for 26 days. 5–6 animals per group were included in the analysis.
